# Mapping multidomain assessment tools for home-visit nursing and rehabilitation: a scoping review

**DOI:** 10.1186/s12913-026-14589-w

**Published:** 2026-04-18

**Authors:** Hirotomo Shibahashi, Kanta Ohno, Tatsunori Sawada

**Affiliations:** Department of Rehabilitation, School of Health Sciences, Tokyo University of Technology, Tokyo, 144-0051 Japan

**Keywords:** Community-dwelling older adults, Home-visit nursing, Interdisciplinary care, Multidomain assessment, Psychometric properties, Scoping review

## Abstract

**Background:**

Multidomain assessment is essential for identifying the complex medical, functional, and psychosocial needs of older adults receiving home care. However, there remains a limited understanding of how existing assessment tools comprehensively address these needs across nursing and rehabilitation disciplines.

**Methods:**

We conducted a date-unrestricted scoping review of PubMed, Scopus, and the Cumulative Index to Nursing and Allied Health Literature (CINAHL) to map assessment tools applicable to home nursing and rehabilitation practices, identifying eight studies published between 2004 and 2024.

**Results:**

The eight included studies employed diverse methodological designs, with each study using a distinct approach, and involved heterogeneous populations and multidomain assessment tools. These tools differed in their scope, conceptual foundations, and psychometric rigor. Most instruments consistently assessed the physical and medical domains, whereas psychosocial health, nutrition, caregiver burden, and patient-reported outcomes were infrequently incorporated. Only a subset of instruments reported evidence of reliability or validity, and the extent and rigor of this evidence varied considerably across studies.

**Conclusions:**

Overall, the findings highlight a fragmented assessment landscape in which existing tools provide valuable but incomplete support for interdisciplinary practices. There is a clear need for an integrated, evidence-informed, multidomain framework tailored to home-visit care to strengthen interdisciplinary communication, enable earlier detection of unmet needs, and enhance the quality of care for community-dwelling older adults. Future research should also address gaps in the availability of validated tools across different languages and cultural contexts.

**Supplementary Information:**

The online version contains supplementary material available at 10.1186/s12913-026-14589-w.

## Background

Rapid population aging has substantially increased the demand for home-visit nursing and community-based rehabilitation services worldwide. In Japan, where population aging is particularly advanced, this demand has grown markedly in recent years [1]. As the clinical complexity of home-dwelling older adults increases, the involvement of rehabilitation professionals, including physical and occupational therapists and speech-language pathologists, has expanded [2]. As patients often present with intertwined medical, functional, psychosocial, and environmental challenges, the delivery of high-quality home-based care increasingly depends on effective coordination among these disciplines [3, 4]. In this context, comprehensive assessment serves as the cornerstone of care planning and early recognition of risks such as functional decline, malnutrition, social vulnerability, and unmet clinical needs [5, 6]. In this study, “home-visit care” refers to healthcare services delivered by nurses and rehabilitation professionals in patients’ homes. The terms “home care” and “home-based care” are used more broadly to encompass various forms of care provided in the home setting, including but not limited to home-visit services. For clarity and consistency, we primarily use the term “home-visit care” when referring to the specific context of this review.

In this context, “domains” denote discrete dimensions of health and functioning (e.g., physical function, psychological status, social participation, and environmental factors), whereas “multidomain” assessments integrate multiple such dimensions to provide a more comprehensive characterization of patients’ complex needs. Several assessment systems currently used in clinical practice, including the Minimum Data Set–Home Care [7], Outcome and Assessment Information Set [8], Functional Independence Measure [9], and Frenchay Activities Index [10], provide meaningful information within their respective domains. Notably, the international Resident Assessment Instrument (interRAI) represents one of the few attempts to establish a comprehensive, standardized framework intended to support information sharing and care planning across multiple care settings and professional disciplines [9]. However, most existing tools were developed for institutional care settings or single professions [8, 9, 11]. Consequently, they frequently emphasize structural or outcome-based metrics rather than capturing the multidimensional profiles of individuals receiving home-visit care, such as the interplay of medical conditions, functional limitations, psychosocial circumstances, and environmental or care-network factors [12, 13]. In addition, many instruments do not adequately reflect the complex and highly individualized nature of the home environment and offer limited support for communication across nursing and rehabilitation disciplines [12, 13]. In the absence of a shared multidomain framework, professionals may rely on discipline-specific instruments that differ in scope, terminology, and thresholds for clinical concern. Such discrepancies may hinder the synthesis of patient information, obscure early signs of deterioration, and constrain the development of coordinated care plans. Consequently, this fragmentation may contribute to inconsistent monitoring, reduced efficiency of interdisciplinary teamwork, and delays in identifying emerging health or functional problems in older adults [14–16].

Despite the recognized importance of integrated assessment in home-visit care, particularly in light of care fragmentation across multiple providers and the complex, interacting health needs associated with multimorbidity and geriatric syndromes among home-dwelling older adults [14, 16], the extent to which existing tools used across disciplines cover domains most relevant to home-dwelling adults with complex needs has not been systematically examined. Existing studies have typically focused on specific instruments or domains rather than providing a comprehensive, cross-disciplinary synthesis. For example, widely used instruments such as the Minimum Data Set–Home Care, Outcome and Assessment Information Set, and Functional Independence Measure have been examined primarily within specific clinical or administrative contexts [7–9], rather than from a comprehensive, multidomain, and cross-disciplinary perspective.

Existing research has not systematically mapped assessment instruments that may be jointly applicable to nurses and rehabilitation professionals, who are key providers in home-visit care and contribute to medical management and functional assessment, respectively [12]. While other professionals (e.g., social workers, psychologists, and midwives) also play important roles in interdisciplinary home-based care, this review specifically focuses on nurses and rehabilitation professionals due to their central role in conducting integrated clinical and functional assessments. The extent to which current tools address critical areas such as nutrition, cognition, psychosocial health, caregiver burden, and patient-reported outcomes is uncertain. Furthermore, the usability, feasibility, and sensitivity of these instruments in real-world home-visit practices have not been comprehensively synthesized.

Given these gaps, there is a pressing need to clarify the landscape of available assessment tools and determine how well they align with the requirements of interdisciplinary home care. In this scoping review, we aimed to identify, categorize, and evaluate assessment instruments that may be applied across nursing and rehabilitation professions in home-visit care. By elucidating the domains covered by existing tools and highlighting areas that remain underrepresented, we aimed to provide an evidence-based foundation for the development of a new, integrated assessment framework that can enhance interdisciplinary collaboration and support high-quality, person-centered care in the home setting.

## Methods

### Study design and protocol registration

This scoping review was conducted in accordance with the methodological framework proposed by the Joanna Briggs Institute (JBI) for scoping reviews and reported following the Preferred Reporting Items for Systematic Reviews and Meta-Analyses extension for Scoping Reviews (PRISMA-ScR) [17, 18]. The protocol was prospectively registered with the Open Science Framework (OSF; DOI: 10.17605/OSF.IO/V8YX7).

### Conceptual framework

The review was guided by the Population, Concept, and Context (PCC) framework recommended by the JBI [19]. The Population comprised adults aged ≥ 18 years, who received home-visit nursing or home-based rehabilitation services. The concept focused on multidomain or comprehensive assessment tools, defined as instruments designed to assess two or more health-related domains, such as medical, functional, psychosocial, and environmental aspects, relevant to home-based care. The context was limited to home-visit nursing, community-based home care, and home rehabilitation settings where nurses, physical therapists, occupational therapists, or speech-language pathologists provided care. This review included only peer-reviewed studies published in English or Japanese and did not consider gray literature. A scoping review approach was selected because the aim of this study was to map the range, characteristics, and domains of existing assessment tools across disciplines, rather than to evaluate the effectiveness of specific interventions. To ensure methodological rigor and consistency of the included evidence, we limited the review to peer-reviewed studies. Although scoping reviews may include a broader range of evidence sources, this restriction was applied to enhance the reliability and interpretability of the findings.

### Eligibility criteria

Studies were included if (1) they involved adults (≥ 18 years) receiving home-visit nursing or home-based rehabilitation; (2) they examined, applied, validated, or described a multidomain assessment tool potentially usable by nursing and rehabilitation professionals; (3) they were published in English or Japanese; and (4) they were peer-reviewed research articles, including original, methodological, and validation studies. To comprehensively identify assessment tools applicable to home-visit care, we included studies involving all adult populations rather than restricting eligibility to older adults alone. This approach was adopted because many assessment instruments are designed for and applied across a broad adult age range.

The exclusion criteria were (1) tools developed exclusively for institutional or acute care settings without applicability to home care; (2) tools assessing a single domain only, such as pain scales, nutritional screens, or activities of daily living (ADL)-specific measures; (3) tools used in studies targeting children or adolescents; (4) tools described in protocol papers, editorials, commentaries, conference abstracts, or studies lacking methodological details; and (5) tools intended solely for physicians, informal caregivers, or administrative personnel, with no applicability to nursing or rehabilitation practice. Tools developed in other disciplines (e.g., psychology or social work) were included if they were considered applicable to nursing or rehabilitation professionals in the context of home-visit care.

### Information sources and search strategy

A comprehensive literature search was conducted using PubMed, Scopus, and the Cumulative Index to Nursing and Allied Health Literature (CINAHL), from database inception to the final search date. The search strategy was developed by the research team through pilot searches, refinement of keywords and indexing terms, and discussion to optimize the balance between sensitivity and specificity. The final strategy incorporated controlled vocabulary (e.g., Medical Subject Headings) and relevant text words related to home-visit nursing, home-based rehabilitation, community-dwelling adults, and assessments. Search strategies were adapted for each database to account for differences in indexing and syntax. The complete search strategies, including all search terms used for each database, are provided in Supplementary Table S1 [see Additional file 1]. No additional manual searches were conducted beyond the electronic database search. For PubMed, multiple complementary search strategies were applied to maximize sensitivity, defined as the ability to capture as many potentially relevant studies as possible, and to ensure comprehensive coverage of relevant concepts. Records retrieved from all searches were combined, and duplicates were removed prior to screening. Search terms related to measurement properties (e.g., validity, reliability, responsiveness) were emphasized in PubMed to enhance specificity for psychometric studies, whereas broader concept-based strategies were applied in Scopus and CINAHL to maximize sensitivity and avoid missing relevant tools not explicitly labeled as validation studies.

### Selection of sources of evidence

Following the database searches, all retrieved records were exported from each database and imported into Microsoft Excel (Microsoft Corporation, Redmond, WA, USA) for deduplication. Duplicate records were then identified and removed prior to screening. Titles and abstracts were independently screened by two reviewers to assess eligibility based on the predefined inclusion and exclusion criteria. Full-text articles were then retrieved and independently assessed for inclusion by the same reviewers. Any disagreements at either stage were resolved through discussion, and when necessary, by consultation with a third reviewer. Screening and data management processes were conducted using Microsoft Excel (Microsoft Corporation, Redmond, WA, USA). No automated screening algorithms were used, and no AI-assisted tools were employed to make inclusion or exclusion decisions during study selection or eligibility assessment. To ensure consistency and quality control, the reviewers conducted pilot screening on a subset of studies prior to full screening. The inclusion criteria were then refined through discussion to clarify definitions and resolve ambiguities. In addition, Study selection was guided by the predefined PCC framework and eligibility criteria, with particular emphasis on identifying assessment tools that addressed multiple health-related domains. Multidomain assessment tools were operationally defined as instruments that explicitly assessed two or more distinct domains of health and functioning, such as physical, psychological, social, or environmental dimensions. Accordingly, studies focusing exclusively on single-domain assessments were excluded.

### Data charting

A standardized data-charting form was developed a priori based on our OSF-registered protocol and refined through team discussion to clarify variables, definitions, and data extraction procedures. The extracted variables included study characteristics (author, year, country, design), target population, sample size, name and purpose of the assessment tool, domains assessed, number of items, administration method, administration time, setting, development process, psychometric properties (reliability, validity, responsiveness), availability of translated versions, usage context, and main findings relevant to home-visit care. Data extraction was performed by one reviewer and verified for accuracy by a second reviewer.

### Synthesis of results

Findings were synthesized descriptively using a narrative approach, consistent with the JBI guidelines. Assessment tools were categorized according to their primary purpose, domain coverage, and applicability across different professional disciplines. Attention was paid to identifying domains that were comprehensively assessed, partially assessed, or underrepresented in the literature. A domain was considered comprehensively assessed when it was explicitly addressed by the majority of identified tools and covered multiple indicators within that domain. Domains addressed by a limited number of tools or represented by a narrow range of indicators were classified as partially assessed, whereas domains infrequently addressed or absent across the identified tools were considered underrepresented. The results are presented using descriptive summaries and tabulated comparisons, offering a clear overview of the current landscape of multidomain assessment tools for home-visit care.

## Results

### Study selection

A total of 421 records were identified through database searches, including 257 from PubMed, 150 from Scopus, and 14 from CINAHL. After removing 15 duplicates, 406 records were screened at the title and abstract levels. Of these, 330 records were excluded for not meeting the eligibility criteria, including being single-domain measures, not constituting multidomain assessment tools, not being applicable to home-visit or community-based care, targeting professionals outside nursing or rehabilitation, or not reporting original research.

**Fig. 1:**
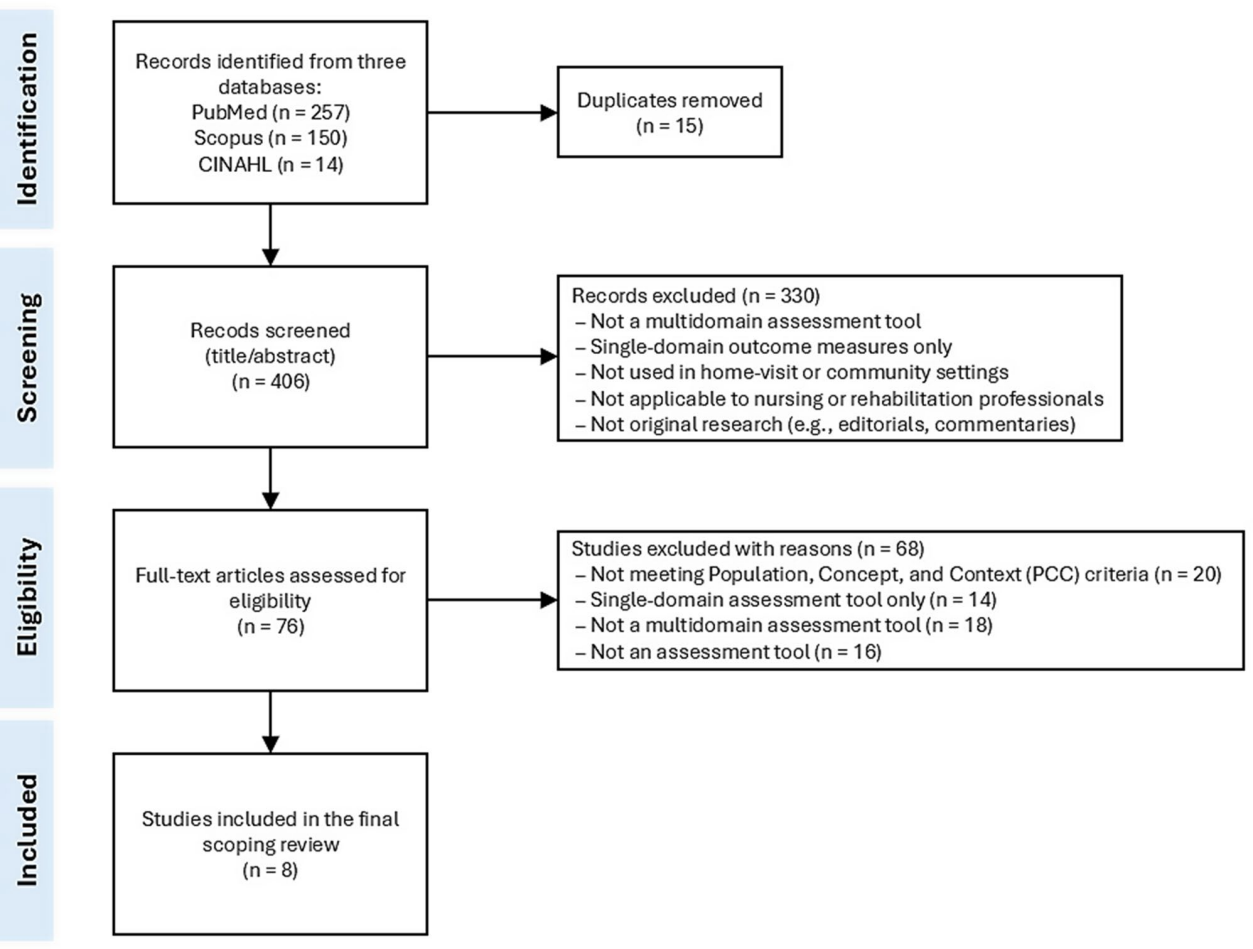
PRISMA flow diagram illustrating the selection process of studies included in the scoping review. This figure outlines the identification, screening, eligibility assessment, and final inclusion of studies

Seventy-six articles underwent full-text review, and 68 were excluded for the following reasons: not meeting the predefined PCC framework (*n* = 20; see Methods, Conceptual Framework and Eligibility Criteria), absence of a comprehensive multidomain assessment instrument (*n* = 18), not constituting an assessment tool (*n* = 16), and assessment of single-domain tools only (*n* = 14). Ultimately, eight studies met all inclusion criteria and were included in the final synthesis. The full study selection process is presented in Fig. 1.

### Study characteristics

The eight studies included in this scoping review were published between 2004 and 2024 and were conducted across multiple countries, including Sweden [20], Norway [21], Thailand [22], New Zealand [23], Japan [24], Brazil [25], Switzerland [26], and a multinational cohort encompassing North America, Europe, and Hong Kong [27]. The included studies involved populations in both home-care settings and community-dwelling contexts (i.e., individuals living independently in the community who may or may not receive formal home-care services), depending on the study design (Supplementary Table S2, see Additional file 1). A summary of the key characteristics and domain coverage of the included assessment tools is presented in Table 1.

**Table 1:** Summary of key characteristics of identified multidomain assessment tools

Tool	Purpose	Domains	Coverage level	Evidence
HAT [20]	Health status stratification	Physical, cognitive, medical, functional	Partial	Predictive validity
SAFE [21]	Early detection of functional decline	Physical, cognitive, functional, symptoms	Partial	Face validity only
ICF-based tool [22]	Multidisciplinary assessment	Physical, functional, participation	Partial	Limited reliability/validity
interRAI HC/SNA [23]	Comprehensive needs assessment	Medical, functional, cognitive, psychosocial, service use	Comprehensive	Extensive prior validation
Preventive MDS–HC [24]	Preventive multidomain assessment	Functional (ADL/IADL), cognitive, psychosocial, environmental	Partial	Limited validity; responsiveness not established
Complexity Assessment Tool [25]	Assessment of care complexity	Clinical and organizational complexity domains	Partial	Good reliability and diagnostic performance
COMID [26]	Multidimensional care complexity assessment	Medical, psychosocial, behavioral, and system-related domains	Comprehensive	Good reliability and validity; responsiveness not established
interRAI IADL–ADL Hierarchy [27]	Functional status scaling	Functional (ADL/IADL)	Partial	Moderate-to-high reliability; construct and predictive validity

ADL, activities of daily living; IADL, instrumental activities of daily living; HAT, Health Assessment Tool; SAFE, Subacute and Acute Dysfunction in the Elderly; ICF, International Classification of Functioning, Disability and Health; interRAI, International Resident Assessment Instrument; HC, Home Care; SNA, Support Needs Assessment, COMID, Complexity of Care in the Home Care Setting Instrument.

The studies employed a broad range of methodological approaches, with each design represented by a single study: a community-based longitudinal cohort study (*n* = 1), a qualitative study (*n* = 1), action research (*n* = 1), a randomized controlled trial (*n* = 1), an observational methodological study for tool development and validation (*n* = 1), a diagnostic accuracy study (*n* = 1), a cross-sectional psychometric evaluation (*n* = 1), and a large-scale secondary data analysis (*n* = 1).

Study populations primarily comprised community-dwelling older adults (*n* = 4), individuals receiving home-care or home-visit services (*n* = 3), and healthcare professionals involved in home-based care (*n* = 1). One large secondary data analysis included mixed populations encompassing both community-based and home-care assessments.

Sample sizes varied substantially across studies, ranging from small qualitative or action-oriented samples with fewer than 50 participants (*n* = 2), to medium-sized cohorts involving approximately 100–300 participants (*n* = 2), larger cohorts of 300–500 participants (*n* = 2), and a single large population-based cohort exceeding 1,000 participants (*n* = 1). In addition, one study involved a very large secondary dataset comprising more than 700,000 assessments.

A total of eight multidomain assessment tools were identified, with each study evaluating a distinct instrument. These included interRAI-related instruments (*n* = 3), as well as Subacute and Acute Dysfunction in the Elderly (SAFE), Health Assessment Tool (HAT), an International Classification of Functioning, Disability, and Health (ICF)-based home-care assessment tool, Complexity Of Care In The Home Care Setting Instrument (COMID), and other complexity or multidomain instruments (each *n* = 1).

Most studies focused on community-dwelling older adults receiving home care or home-visit services, many of whom present with multimorbidity, functional limitations, or emerging care needs. Sample sizes varied widely, from 10 participants in a qualitative study of Norwegian home care nurses [21] to more than 760,000 interRAI assessments in an international secondary analysis [27]. Several studies evaluated assessment tools directly during home visits, whereas others derived or validated instruments using existing home care assessment records.

Across the eight studies, the assessment tools were administered by various professionals, including home care nurses, public health nurses, physical therapists, multidisciplinary home care teams, and trained assessors.

### Overview of identified assessment tools

Eight multidomain assessment tools applicable to home-visit nursing and rehabilitation practices were identified in this review (Supplementary Table S3, see Additional file 1). These instruments exhibit substantial variations in their scope, intended clinical purpose, underlying conceptual frameworks, and degree of comprehensiveness. The identified tools were administered in the respective local languages of the study settings; most included articles were published in English, with one study published in Japanese.

HAT integrates indicators of gait speed, cognitive performance, chronic disease burden, and ADL/instrumental activities of daily living (IADL) limitations to derive a hierarchical measure of overall health status and predict the subsequent utilization of health and social care services among community-dwelling older adults [20]. SAFE is a structured observational checklist comprising 13 domains, including communication, elimination, nutrition, physical function, and self-care, designed to facilitate the early detection of emerging functional decline during routine home care visits [21]. An ICF-based Home Health Care Assessment Tool developed in Thailand incorporates 12 ICF categories to guide multidisciplinary assessment and individualized home care planning [22].

The interRAI Home Care instrument and its preventive adaptations of the Minimum Data Set–Home Care provide comprehensive coverage of the medical, functional, cognitive, psychosocial, and environmental domains [23, 24]. These tools are embedded within a decision-support system that generates clinical assessment protocols and supports structured needs assessments for preventive home visits. The Brazilian complexity assessment tool captures clinical and organizational complexities by evaluating home and managerial activities relevant to care planning [25]. COMID assesses six domains of care complexity—medical, social, mental health, behavioral, instability-related, and system-level factors—and was recently validated in an Italian version for home care nurses [26]. Finally, the interRAI IADL hierarchy scale and interRAI IADL–ADL functional hierarchy offer standardized measures of functional status across interRAI instruments, enabling consistent stratification of dependence in community and home care contexts [27].

The reporting of psychometric properties across studies was uneven. Several instruments, including the interRAI functional hierarchies and COMID, have demonstrated acceptable internal consistency, strong interrater reliability, and evidence of construct or predictive validity [26, 27]. In contrast, tools such as SAFE and ICF-based assessments are supported primarily by user acceptability and perceived clinical utility rather than rigorous psychometric evaluations [21, 22]. Evidence regarding the responsiveness to change and feasibility of routine home-visit workflows remains limited. Notably, while most tools addressed physical and medical domains, relatively few incorporated nutrition, caregiver burden, or patient-reported outcomes, highlighting persistent gaps in the comprehensiveness of multidomain assessments used in home care settings.

## Discussion

In this scoping review, we identified eight multidomain assessment tools applicable to home-visit nursing and rehabilitation practices. Collectively, these studies illustrate how multidomain assessments are conceptualized and implemented across different health systems and service delivery models, providing a comprehensive overview of the approaches currently used to evaluate the complex needs of individuals receiving home-visit care. Although the tools differed substantially in their clinical purpose, conceptual foundations, and level of comprehensiveness, most were designed to support the evaluation of complex needs among community-dwelling older adults. Functional and medical domains were consistently represented, whereas psychosocial, nutritional, and caregiver-related domains were incorporated less frequently. The reporting of psychometric properties was variable, with only a subset of tools reporting evidence of reliability or validity. Overall, the findings reveal a fragmented landscape in which existing assessment instruments offer valuable but incomplete approaches to guide interdisciplinary home-visit care. From a conceptual perspective, the distinction between single-domain and multidomain assessments is central to understanding the implications of these findings. Single-domain instruments, such as those focusing exclusively on physical function or cognition, offer depth within a specific area but may fail to capture the complex, interacting needs of individuals receiving home-visit care. In contrast, multidomain assessments aim to provide a more comprehensive evaluation by integrating multiple dimensions of health and functioning. However, as demonstrated in this review, many existing tools only partially achieve this integration, often emphasizing selected domains while omitting others. This suggests that the distinction between single-domain and multidomain approaches is not binary but exists along a continuum, with important implications for both clinical practice and tool development.

The fragmented nature of multidomain assessment observed in this review is consistent with findings from earlier literature on home visits and community-based geriatric care [28]. Previous studies have highlighted that most existing assessment frameworks were originally developed within institution-based or single-profession models, limiting their utility for interdisciplinary practice in the home setting [29, 30]. Similar to our findings, earlier reviews of interRAI instruments and preventive home-visit programs have demonstrated strong coverage of medical and functional domains, but noted substantial variability in the integration of psychosocial, environmental, and caregiver-related factors [28, 31]. Moreover, studies examining early functional decline detection tools such as SAFE [21], have emphasized their clinical value despite limited psychometric validation, echoing the methodological inconsistencies observed in this review. These findings confirm that although several tools provide important contributions, no existing instrument fully meets the multidimensional and interdisciplinary requirements of contemporary home visit care.

Several factors may explain the fragmented development of multidomain assessment tools for home-visit care across different healthcare systems. In many countries, home-based services have historically evolved within parallel professional streams (e.g., nursing, rehabilitation, and social care), resulting in the development of assessment instruments that reflect discipline-specific priorities rather than shared interdisciplinary needs. In Japan, although policy-level integration has been promoted since the introduction of the long-term care insurance system in 2000, previous studies suggest that service delivery and assessment practices often remain structured around profession-specific frameworks rather than fully integrated models [32, 33]. Consistent with the findings of this review, these structural challenges are likely not unique to Japan but may also be observed in other health systems undergoing transitions toward integrated, community-based care. These gaps have important clinical implications: the absence of a unified assessment framework hampers communication across professions, reduces the efficiency of care planning, and increases the risk of overlooking psychosocial, nutritional, and caregiver-related issues that influence health trajectories. Developing an integrated, multidomain tool tailored to the realities of home-visit care may enhance interdisciplinary coordination and improve the early identification of unmet needs among community-dwelling older adults.

This review has some limitations. First, although we conducted a comprehensive search across three major databases, relevant studies indexed elsewhere or published in languages other than English or Japanese may not have been captured, potentially introducing selection bias. Second, substantial methodological heterogeneity among the included studies precluded direct comparison of psychometric properties across instruments. Moreover, the limited availability of rigorous validation studies for several tools restricted evaluation of their measurement quality. Third, as a scoping review, our objective was to map available assessment tools rather than assess their effectiveness; therefore, conclusions regarding clinical utility should be interpreted with caution. Finally, by focusing on multidomain instruments aligned with the predefined PCC framework, we may have excluded single-domain tools that are used in combination in clinical practice.

## Conclusions

This scoping review identified substantial fragmentation among existing multidomain assessment tools used in home-visit care, reflecting their development within profession-specific frameworks. While these tools vary in scope and purpose, few were designed to support truly integrated, interdisciplinary assessment. Based on these findings, the development of an integrated, evidence-informed multidomain assessment tool tailored to home-visit care is warranted. Such a tool has the potential to enhance interdisciplinary communication, facilitate early identification of unmet needs, and ultimately improve the quality of care for older adults living at home. Future research should focus on the co-design, validation, and implementation of assessment frameworks aligned with routine home-visit practice across professional disciplines. In addition, given the limited availability of validated multidomain assessment tools in certain languages, particularly Japanese, as identified in this review, future studies should prioritize their development and cross-cultural adaptation to ensure applicability across diverse linguistic and healthcare contexts.

## Supplementary Information

Below is the link to the electronic supplementary material.

Supplementary Material 1

## Data Availability

All data generated or analyzed in this study were derived from the published literature. The dataset supporting the findings of this review is available from the corresponding author upon reasonable requests.

## Abbreviations

ADL: Activities of daily living
CINAHL: Cumulative Index to Nursing and Allied Health Literature
COMID: Complexity Of Care In The Home Care Setting Instrument
HAT: Health Assessment Tool
IADL: Instrumental activities of daily living
ICF: International Classification of Functioning, Disability, and Health
interRAI: International Resident Assessment Instrument
JBI: Joanna Briggs Institute
OSF: Open Science Framework
PCC: Population, Concept, and Context
PRISMA-ScR: Preferred Reporting Items for Systematic Reviews and Meta-Analyses extension for Scoping Reviews
SAFE: Subacute and Acute Dysfunction in the Elderly
