# Supplementary material for: Mapping multidomain assessment tools for home-visit nursing and rehabilitation: a scoping review
Source: BMC Health Serv Res. 2026 Apr 18;26:775. doi: 10.1186/s12913-026-14589-w (PMC13224622; doi:10.1186/s12913-026-14589-w)
Supplement: Supplementary file 1 — Supplementary Material 1 [file 12913_2026_14589_MOESM1_ESM.docx]

**Supplementary Table S1**

**Full Search Strategies for Each Database**

This supplementary file provides the complete search strategies used for each database to ensure transparency and reproducibility of the scoping review, in accordance with the PRISMA-ScR guidelines.

**PubMed**

The PubMed search was conducted iteratively using multiple search strategies that were refined to maximize sensitivity and relevance.

Database: PubMed

Platform: National Library of Medicine

|  | Search strategy |
| --- | --- |
| Search 1 (initial exploratory search) | (("home care services" OR "home health nursing" OR "visiting nursing" OR "home rehabilitation" OR "home physical therapy" OR "home occupational therapy") AND ("assessment tool" OR "measurement instrument" OR "questionnaire" OR "evaluation scale") AND ("validation studies" OR "psychometric properties" OR "reliability" OR "validity" OR "responsiveness" OR "interpretability")) |
| Search 2 (expanded MeSH-based search) | ( ("Home Care Services"[MeSH] OR "Community Health Nursing"[MeSH] OR "Home Nursing"[TIAB] OR "Home Health Care"[TIAB] OR "Domiciliary Care"[TIAB] OR "Home-Based Care"[TIAB] OR "Home Visit Nursing"[TIAB]) ) AND ( ("Nursing"[MeSH] OR "Nurses"[MeSH] OR "Nursing Care"[MeSH] OR "Nurse"[TIAB] OR "Home Nurses"[TIAB] OR "Community Nurses"[TIAB]) ) AND ( ("Rehabilitation"[MeSH] OR "Physical Therapy Modalities"[MeSH] OR "Occupational Therapy"[MeSH] OR "Physiotherapy"[TIAB] OR "Physical Therapy"[TIAB] OR "Occupational Therapy"[TIAB] OR "Therapists"[TIAB] OR "Rehabilitation Services"[TIAB]) ) AND ( ("Outcome Assessment, Health Care"[MeSH] OR "Patient Reported Outcome Measures"[MeSH] OR "Evaluation Tool"[TIAB] OR "Assessment Tool"[TIAB] OR "Functional Assessment"[TIAB] OR "Multidisciplinary Assessment"[TIAB] OR "Comprehensive Assessment"[TIAB]) ) AND ( ("Interprofessional Relations"[MeSH] OR "Patient Care Team"[MeSH] OR "Collaborative Care"[TIAB] OR "Multidisciplinary Team"[TIAB] OR "Interdisciplinary Care"[TIAB] OR "Team-Based Care"[TIAB] OR "Interprofessional Collaboration"[TIAB]) ) |
| Search 3 (expanded MeSH-based search) | ( "home care"[TIAB] OR "home nursing"[TIAB] OR "home health care"[TIAB] OR "domiciliary care"[TIAB] OR "community nursing"[TIAB] ) AND ( "nurse"[TIAB] OR "nurses"[TIAB] OR "nursing care"[TIAB] OR "physical therapy"[TIAB] OR "occupational therapy"[TIAB] OR "rehabilitation"[TIAB] ) AND ( "evaluation tool"[TIAB] OR "assessment tool"[TIAB] OR "functional assessment"[TIAB] OR "clinical assessment"[TIAB] ) |
| Search 4 (expanded MeSH-based search) | (("Home Care Services"[MeSH] OR "home health care"[TIAB] OR "home care"[TIAB] OR "community nursing"[TIAB])) AND (("Nurses"[MeSH] OR "Nursing"[MeSH] OR "nurse"[TIAB] OR "nurses"[TIAB] OR "nursing care"[TIAB])) AND (("Physical Therapy Modalities"[MeSH] OR "Rehabilitation"[MeSH] OR "Occupational Therapy"[MeSH] OR "Physical Therapists"[MeSH] OR "Occupational Therapists"[MeSH] OR "physical therapy"[TIAB] OR "occupational therapy"[TIAB] OR "rehabilitation"[TIAB] OR "physiotherapy"[TIAB] OR "therapist"[TIAB]))  AND (("Health Status Indicators"[MeSH] OR "Health Surveys"[MeSH] OR "Patient Reported Outcome Measures"[MeSH] OR "Outcome Assessment, Health Care"[MeSH] OR "assessment tool"[TIAB] OR "evaluation tool"[TIAB] OR "clinical assessment"[TIAB] OR "functional assessment"[TIAB] OR "multidisciplinary assessment"[TIAB] OR "comprehensive assessment"[TIAB])) AND (("Patient Care Team"[MeSH] OR "Interprofessional Relations"[MeSH] OR "multidisciplinary team"[TIAB] OR "interprofessional teamwork"[TIAB] OR "integrated care"[TIAB] OR "team-based care"[TIAB])) AND (("Review"[Publication Type] OR "Scoping Review"[TIAB] OR "Systematic Review"[TIAB] OR "Literature Review"[TIAB])) |
| Search 5　(expanded MeSH-based search) | (("Home Care Services"[MeSH] OR "Community Health Nursing"[MeSH] OR "Home Nursing"[TIAB] OR "Home Health Care"[TIAB] OR "Domiciliary Care"[TIAB] OR "Home-Based Care"[TIAB] OR "Home Visit Nursing"[TIAB])) AND (("Nursing"[MeSH] OR "Nurses"[MeSH] OR "Nursing Care"[MeSH] OR "Nurse"[TIAB] OR "Home Nurses"[TIAB] OR "Community Nurses"[TIAB])) AND (("Rehabilitation"[MeSH] OR "Physical Therapy Modalities"[MeSH] OR "Occupational Therapy"[MeSH] OR "Physiotherapy"[TIAB] OR "Physical Therapy"[TIAB] OR "Occupational Therapy"[TIAB] OR "Therapists"[TIAB] OR "Rehabilitation Services"[TIAB])) AND (("Outcome Assessment, Health Care"[MeSH] OR "Patient Reported Outcome Measures"[MeSH] OR "Evaluation Tool"[TIAB] OR "Assessment Tool"[TIAB] OR "Functional Assessment"[TIAB] OR "Multidisciplinary Assessment"[TIAB] OR "Comprehensive Assessment"[TIAB])) AND (("Interprofessional Relations"[MeSH] OR "Patient Care Team"[MeSH] OR "Collaborative Care"[TIAB] OR "Multidisciplinary Team"[TIAB] OR "Interdisciplinary Care"[TIAB] OR "Team-Based Care"[TIAB] OR "Interprofessional Collaboration"[TIAB])) AND (("Review"[Publication Type] OR "Scoping Review"[TIAB] OR "Systematic Review"[TIAB] OR "Narrative Review"[TIAB] OR "Literature Review"[TIAB])) |

**Scopus**

Database: Scopus

Provider: Elsevier

Search strategy:

|  | Search strategy |
| --- | --- |
| Search 1 | ("home care" OR "home health care" OR "home-based care" OR "domiciliary care" OR "home visit nursing" OR "community nursing") AND ("nurse" OR "nursing care" OR "physical therapy" OR "occupational therapy" OR "rehabilitation") AND ("assessment tool" OR "evaluation tool" OR "questionnaire" OR "functional assessment") AND ("interprofessional collaboration" OR "patient care team" OR "team-based care" OR "multidisciplinary team") |

**CINAHL**

Database: CINAHL

Platform: EBSCOhost

Search strategy:

|  | Search strategy |
| --- | --- |
| Search 1 | ((MH "Home Care Services+" OR MH "Home Health Care+" OR MH "Community Health Nursing+" OR TX ("home care" OR "home health care" OR "home-based care" OR "home nursing" OR "visiting nursing" OR "domiciliary care" OR "community nursing" OR "home rehabilitation" OR "home physical therapy" OR "home occupational therapy")) AND (MH "Nurses+" OR MH "Nursing Care+" OR MH "Rehabilitation+" OR MH "Physical Therapy+" OR MH "Occupational Therapy+" OR TX ("nurse" OR "nursing care" OR "rehabilitation" OR "physical therapy" OR "occupational therapy" OR "physiotherapy" OR "therapist")) AND (MH "Outcome Assessment (Health Care)+" OR MH "Health Status Indicators+" OR MH "Health Surveys+" OR MH "Patient Reported Outcome Measures+" OR TX ("assessment tool" OR "evaluation tool" OR "measurement tool" OR "questionnaire" OR "functional assessment" OR "comprehensive assessment" OR "multidisciplinary assessment")) AND (MH "Interprofessional Relations+" OR MH "Patient Care Team+" OR TX ("interprofessional collaboration" OR "multidisciplinary team" OR "team-based care" OR "interdisciplinary care" OR "collaborative care")) AND TX ("validation" OR "psychometric" OR "reliability" OR "validity" OR "responsiveness" OR "interpretability" OR "scale development" OR "instrument development") ) AND (TI ("validation" OR "psychometric" OR "scale" OR "instrument" OR "questionnaire") OR AB ("validation" OR "psychometric" OR "scale" OR "instrument" OR "questionnaire")) NOT TX ("hospital" OR "acute care" OR "pediatric" OR "child" OR "pain scale" OR "nutrition" OR "sleep") |

**Supplementary Table S2**. Characteristics of included studies (n = 8)

| No. | First author (year) | Country | Study design | Population / setting | Sample size* | Assessment tool(s) |
| --- | --- | --- | --- | --- | --- | --- |
| 1 | Santoni et al. [20] | Sweden | Community-based longitudinal cohort (SNAC-K) | Community-dwelling adults ≥65 years in an urban Swedish district | 2,179 | Health Assessment Tool (HAT) derived from integrated clinical and functional assessment data |
| 2 | Abudayya et al. [21] | Norway | Qualitative study (individual interviews and focus groups) | Home-care work team coordinators (nurses) using SAFE in home-based nursing | 10 | SAFE (Subacute and Acute Dysfunction in the Elderly) |
| 3 | Pimdee & Nualnetr [22] | Thailand | Action research (three-phase implementation project) | Home health-care team (nurses, physical therapists, traditional medicine practitioners) and home-bound patients | 10 health-care staff; 30 home-bound patients | International Classification of Functioning, Disability, and Health (ICF)-based Home Health Care Assessment Tool (12-category ICF-based instrument) |
| 4 | Parsons et al. [23] | New Zealand | Randomized controlled trial (interRAI-HC vs Support Needs Assessment) | Community-dwelling adults aged ≥65 years referred for comprehensive needs assessment in New Zealand | 316 | interRAI Home Care (HC) instrument and Support Needs Assessment (SNA) |
| 5 | Yamada & Ikegami [24] | Japan | Observational methodological study for tool development and validation | Community-dwelling older adults with IADL limitations targeted for preventive home visits | Sample: 217 (primary analysis); 163 (longitudinal subset) | Preventive version of the MDS–HC (89-item multidomain assessment) |
| 6 | Bôas et al. [25] | Brazil | Diagnostic-accuracy study | Home-care patients with chronic diseases and functional limitations | 253 | Complexity Assessment Tool (20 home and managerial activities) |
| 7 | Levati et al. [26] | Switzerland (Italian-speaking region) | Cross-sectional psychometric evaluation | Older adults receiving home-care services; assessments completed by home-care nurses and MSc nursing students | 110 patients (220 COMID ratings) | COMID (Complexity of Care in the Home Care Setting Instrument), Italian version |
| 8 | Morris et al. [27] | Multinational (North America, Europe, Hong Kong) | Secondary analysis of routine assessment data | Older adults receiving home-care or community assessments within the interRAI system | 762,023 assessments | interRAI IADL Hierarchy Scale and interRAI IADL–ADL Functional Hierarchy |

*Sample size refers to the primary analytical sample reported for each study.

*“Population/setting” refers to the target population and care context, including home-care settings and community-dwelling populations (i.e., individuals living independently in the community, with or without formal home-care services).

**Supplementary Table S3**. Overview of identified multidomain assessment tools

| Tool (abbreviation) | Study | Primary purpose | Target population / setting | Key health indicators assessed | Approx. no. of items / indicators | Administrator(s) | Language of administration | Reported psychometric properties |
| --- | --- | --- | --- | --- | --- | --- | --- | --- |
| Health Assessment Tool (HAT) | Santoni et al. [20] | Stratification of overall health status and associated health- and social-care use | Community-dwelling older adults ≥65 years | Gait speed, cognitive function (MMSE), number of chronic diseases, IADL and ADL limitations | Five indicators | Trained health professionals using validated clinical and functional assessments | Swedish | Component measures previously validated; clear dose–response relationship between HAT levels and subsequent service utilization (predictive validity); no formal reliability or responsiveness analyses for the composite score |
| SAFE (Subacute and Acute Dysfunction in the Elderly) | Abudayya et al. [21] | Early detection of subacute or acute functional decline in home-care recipients | Older adults receiving home-based nursing care | Thirteen observational domains including communication, elimination, nutrition, physical function, general self-care, personal hygiene, medical self-care, sleep, cognitive function, and pain | Thirteen domains with color-coded risk signals | Home-care nurses conducting structured observations during visits | Norwegian | No quantitative reliability data; users reported that SAFE was clinically meaningful and supported decision-making (face validity). responsiveness not evaluated |
| ICF-based Home Health Care Assessment Tool | Pimdee & Nualnetr [22] | Structuring multidisciplinary assessment and care planning using the ICF framework | Home-bound adults living in the community and receiving municipal home health care in Thailand | Twelve ICF categories (pain, joint mobility, muscle power, muscle endurance, mobility, walking, changing and maintaining body position, self-care, domestic life, and community participation) | Twelve ICF categories with standard qualifiers (0–4) | Trained research assistant administering the assessment during home visits; findings reviewed by a multidisciplinary home health care team in case conferences | Thai | Internal consistency of associated staff and patient questionnaires acceptable (Cronbach’s α ≈ 0.74–0.84); formal reliability and validity testing of the ICF tool itself limited; qualitative evidence that changes in ICF categories paralleled clinical improvement |
| interRAI Home Care (HC) and Support Needs Assessment (SNA) | Parsons et al. [23] | Comprehensive needs assessment and identification of unmet needs, including triggering of Clinical Assessment Protocols (CAPs), for community-dwelling older adults | Community-dwelling adults aged ≥65 years referred for comprehensive needs assessment in New Zealand | Wide range of domains including medical conditions, function, cognition, mood, behavior, social support, and service use | Full interRAI-HC: >200 items; SNA shorter structured assessment | Registered health professionals conducting standardized home assessments | English | interRAI instruments supported by extensive prior validation; in this trial, interRAI-HC improved identification of needs but did not increase service provision or short-term outcomes; no new psychometric estimates reported |
| Preventive MDS–HC (89-item version) | Yamada & Ikegami [24] | Development of a shorter multidomain tool to structure preventive home visits and trigger CAPs | Community-dwelling older adults with emerging IADL problems in Japan | Multiple domains including ADL, IADL, cognition, mood, social support, and environmental risks | Reduced from 247 to 89 items | Public health nurses conducting home visits every 3 months | Japanese | Item reduction based on observational data; some CAPs showed limited sensitivity; external validity and responsiveness not fully established |
| Complexity Assessment Tool (home care) | Bôas et al. [25] | Quantification of clinical and organizational complexity in patients receiving home care | Adults with chronic conditions followed in Brazilian home-care services | Home activities and managerial activities reflecting care complexity (20 activities) | 20 activities scored by time and complexity | Two independent examiners performing home visits and repeated ratings | Portuguese | Demonstrated acceptable interrater reliability and diagnostic performance (sensitivity, specificity, ROC analyses, kappa statistics); results supported its use as a screening and monitoring tool for complex home-care cases |
| COMID (Complexity of Care in the Home Care Setting Instrument), Italian version | Levati et al. [26] | Assessment of multidimensional care complexity to support clinical judgement and care planning | Older adults receiving home-care services in Italian-speaking Switzerland | Six factors: medical health, social and economic factors, mental health, patient behavior, instability, and care provider/system factors | Thirty dichotomous items (yes/no; total score 0–30) | Home-care nurses and MSc nursing students independently rating the same patients | Italian | Good internal consistency (KR-20 ≈ 0.76 overall); excellent interrater reliability for total and subscale scores (ICC 0.85–0.97); substantial to excellent agreement at item level (Cohen’s kappa 0.67–0.93); evidence of face and content validity based on clinical utility; responsiveness not yet assessed |
| interRAI IADL Hierarchy Scale and IADL–ADL Functional Hierarchy | Morris et al. [27] | Standardized scaling of functional status across the interRAI suite of instruments | Older adults assessed in home-care and community settings within interRAI programs | Five IADL items (meal preparation, housework, shopping, managing finances, medication management) and four ADL items (hygiene, locomotion, toilet use, eating) | Nine items combined into hierarchical scales | Trained interRAI assessors completing routine interRAI HC or CHA assessments | English | Internal consistency was moderate to high (KR-20 = 0.62–0.86 across countries). Functional scales are strongly associated with hours of formal and informal care and cognitive performance (construct and predictive validity). responsiveness not examined |

Abbreviations: ADL, activities of daily living; IADL, instrumental activities of daily living; HAT, health assessment tool; ICF, International Classification of Functioning, Disability, and Health; SAFE, Subacute and Acute Dysfunction in the Elderly; ICC, intraclass correlation coefficient; HC, home care; CHA, community health assessments
